# Spatial and temporal variation in the *kdr *allele L1014S in *Anopheles gambiae *s.s. and phenotypic variability in susceptibility to insecticides in Western Kenya

**DOI:** 10.1186/1475-2875-10-10

**Published:** 2011-01-14

**Authors:** Derrick K Mathias, Eric Ochomo, Francis Atieli, Maurice Ombok, M Nabie Bayoh, George Olang, Damaris Muhia, Luna Kamau, John M Vulule, Mary J Hamel, William A Hawley, Edward D Walker, John E Gimnig

**Affiliations:** 1 Centre for Global Health Research, Kenya Medical Research Institute, PO Box 1578, Kisumu, Kenya; 2 Centers for Disease Control and Prevention, PO Box 1578, Kisumu, Kenya; 3 Centre for Biotechnology Research and Development, Kenya Medical Research Institute, P.O Box 54840 - 00200, Nairobi, Kenya; 4 Division of Parasitic Diseases, Centers for Disease Control and Prevention, 4770 Buford Hwy, Mailstop F-42, Atlanta, GA, 30341, USA; 5 Department of Microbiology and Molecular Genetics, Michigan State University, East Lansing, MI, 48824, USA; 6 Department of Molecular Microbiology and Immunology, Johns Hopkins Bloomberg School of Public Health, 615 N Wolfe St, Baltimore, MD, 21205, USA

## Abstract

**Background:**

Malaria vector control in Africa depends upon effective insecticides in bed nets and indoor residual sprays. This study investigated the extent of insecticide resistance in *Anopheles gambiae *s.l., *Anopheles gambiae *s.s. and *Anopheles arabiensis *in western Kenya where ownership of insecticide-treated bed nets has risen steadily from the late 1990s to 2010. Temporal and spatial variation in the frequency of a *knock down resistance *(*kdr*) allele in *A. gambiae *s.s. was quantified, as was variation in phenotypic resistance among geographic populations of *A. gambiae *s.l.

**Methods:**

To investigate temporal variation in *kdr *frequency, individual specimens of *A. gambiae *s.s. from two sentinel sites were genotyped using RT-PCR from 1996-2010. Spatial variation in *kdr *frequency, species composition, and resistance status were investigated in additional populations of *A. gambiae *s.l. sampled in western Kenya in 2009 and 2010. Specimens were genotyped for *kdr *as above and identified to species via conventional PCR. Field-collected larvae were reared to adulthood and tested for insecticide resistance using WHO bioassays.

**Results:**

*Anopheles gambiae *s.s. showed a dramatic increase in *kdr *frequency from 1996 - 2010, coincident with the scale up of insecticide-treated nets. By 2009-2010, the *kdr *L1014S allele was nearly fixed in the *A. gambiae *s.s. population, but was absent in *A. arabiensis*. Near Lake Victoria, *A. arabiensis *was dominant in samples, while at sites north of the lake *A. gambiae *s.s was more common but declined relative to *A. arabiensis *from 2009 to 2010. Bioassays demonstrated that *A. gambiae *s.s. had moderate phenotypic levels of resistance to DDT, permethrin and deltamethrin while *A. arabiensis *was susceptible to all insecticides tested.

**Conclusions:**

The *kdr *L1014S allele has approached fixation in *A. gambiae *s.s. populations of western Kenya, and these same populations exhibit varying degrees of phenotypic resistance to DDT and pyrethroid insecticides. The near absence of *A. gambiae *s.s. from populations along the lakeshore and the apparent decline in other populations suggest that insecticide-treated nets remain effective against this mosquito despite the increase in *kdr *allele frequency. The persistence of *A. arabiensis*, despite little or no detectable insecticide resistance, is likely due to behavioural traits such as outdoor feeding and/or feeding on non-human hosts by which this species avoids interaction with insecticide-treated nets.

## Background

Malaria vector control programmes in sub-Saharan Africa continue to rely heavily on indoor residual spraying (IRS) or insecticide-treated nets (ITNs), both of which depend on vector susceptibility to the insecticides used. ITNs are a common component of malaria control programs, largely due to their ease of implementation, cost effectiveness [1-3], and record of success across transmission settings [4]. As a consequence, ITNs have been heavily promoted by the public-health community; and as of 2010, it was estimated that 42% of households owned at least one ITN across 44 countries in sub-Saharan Africa [5]. These advances in vector control, combined with effective new anti-malarial drugs, have renewed optimism for regional elimination [6,7].

Few insecticides are available for use in malaria vector control and only pyrethroid compounds are considered safe for the treatment of ITNs [8]. Therefore, the continued success of ITNs and essentially the current vector-control paradigm depend on continued mosquito susceptibility to a single class of insecticides. Two mechanisms of insecticide resistance, which may co-occur, are commonly measured: (1) metabolic resistance and (2) target-site insensitivity [9]. The former results from the over expression or amplification of genes coding for enzymes that either biochemically alter the insecticidal compound making it less toxic to the mosquito (e.g. P450 monooxygenases), or sequester the compound preventing reactions detrimental to normal physiology (e.g. esterases) [9,10]. In contrast, target-site insensitivity involves one or more mutations that make the physiologic target of an insecticide less reactive to the chemical [11]. Target-site insensitivity in the malaria vector *Anopheles gambiae *s.s. (hereafter referred to as *A. gambiae*) includes two *kdr *mutations (for knock-down resistance) which result in an amino acid substitution in the S6 hydrophobic segment of domain II in the voltage-gated sodium channel of neuronal membranes [9,11]. Both mutations reduce susceptibility to DDT and to the pyrethroid class of insecticides and occur at different nucleotides of the same amino acid (residue 1014). In wild-type mosquitoes, this residue codes for leucine (TTA), while in mutant mosquitoes single nucleotide changes result in either a phenylalanine (TTT) or serine (TCA) substitution [12,13]. The former is commonly referred to as West African *kdr*, or L1014F; while the latter is called East African *kdr*, or L1014S. Molecular data suggest that each mutation has risen independently at least twice in *A. gambiae *[14], and, although the names of each allele suggest distinct geographic distributions, co-occurrence has been reported in multiple countries including Uganda, Gabon, Cameroon, Equatorial Guinea, and Angola [15]. L1014F is thought to confer a greater degree of insensitivity to pyrethroid insecticides than L1014S [11], although bioassay data comparing each mutation within the same genetic background do not exist. Nevertheless, it has been speculated that selection for L1014F should be stronger than for L1014S, a hypothesis supported by recent molecular data showing that signatures of selective sweeps (i.e. reduced nucleotide variability) are more extensive around L1014F than L1014S mutations in the genomes of wild-caught mosquitoes [16]. A third mechanism of resistance--behavioural--is poorly understood and difficult to measure, although the excitatory effects of DDT in IRS and permethrin in certain ITN formulations suggest that a primary mode of action of these two chemicals is on vector behaviour and not survival [17-20].

When monitoring or investigating insecticide resistance in vector populations, at least three approaches can be taken, each with advantages and disadvantages: (1) measures of phenotypic resistance provide a direct indication of how resistance mechanisms impact vector control activities but require access to testing kits, insecticide impregnated papers, rearing facilities, and large numbers of mosquitoes, any of which may be limiting; (2) frequencies of target site mutations (e.g. *kdr*) are easier to measure, but it is unclear how much this mechanism contributes to resistant phenotypes; (3) measures of metabolic resistance (e.g. biochemical assays or gene expression arrays) are likely to be strong indicators of phenotypic resistance but are technically challenging.

Malaria vectors in western Kenya, where the present study was conducted, include two sibling species of the *A. gambiae *species complex, *A. gambiae *and *Anopheles arabiensis*, as well as *Anopheles funestus *s.s. Historically, *A. gambiae *has been the primary vector in the study area, but this species has declined proportionately to *A. arabiensis *(a secondary vector), possibly due to greater effectiveness of ITNs against the former, more anthropophilic species [21]. Development of insecticide resistance in the local *A. gambiae *population could reverse this trend and might reduce effectiveness of the malaria control program in the region.

Two of the study sites included in the present study are within or adjacent to areas included in a small ITN trial conducted in 1990 and a large-scale trial conducted from 1996 to 1999. The former involved just six villages [22], while the latter covered a 500 km^2 ^area that encompassed 221 villages [23]. In the smaller trial, *A. gambiae *mosquitoes collected from villages with permethrin-treated nets and curtains showed increased tolerance to permethrin after one year, leading to establishment of a laboratory strain with reduced sensitivity to permethrin [24]. However, this tolerance did not persist in field populations, and required constant selection in the laboratory to maintain the phenotype [25]. The tolerant field population had increased activity of metabolic enzymes as well as a novel mutation in the sodium channel gene (i.e. *kdr *L1014S) [13,26]. Studies of insecticide resistance following the larger ITN trial have varied with respect to both levels of phenotypic resistance and frequency of the *kdr *L1014S mutation. In 2004, Stump et al. [27] reported that *kdr *frequency was 8.0% in *A. gambiae *and had only risen slightly since 1987. A more recent study reported a range of 0.5% to 15.0% among sites in western Kenya for *A. gambiae *and 0.9% for *A. arabiensis*, but "conservative" population estimates for the former were given as 1.0% or less when accounting for relatedness of the specimens [28]. That study also reported an increase in monooxygenase activity in field-collected specimens. Another recent study reported a *kdr *L1014S frequency of 25.4% in a sub-population of *A. gambiae *from western Kenya [29]. WHO bioassays, however, provided no evidence of phenotypic resistance. Whether genotypic or phenotypic insecticide resistance is evolving in the *A. gambiae *population in direct response to the rapid rise in use of ITNs in western Kenya, in parallel with the profound changes in species composition of the *A. gambiae *complex there [21], is currently unknown. Therefore, the objective of this study was to quantify temporal variation in *kdr *L1014S frequency in populations of *A. gambiae *(1996 - 2010) from two areas of western Kenya having markedly different histories of ITN distribution and use; and to assess the spatial variation in frequency of the *kdr *L1014S allele, species composition, and phenotypic resistance among populations of *A. gambiae *s.l. sampled from multiple sites in western Kenya.

## Methods

### Temporal variation in *kdr *allele frequency

**Figure 1:**
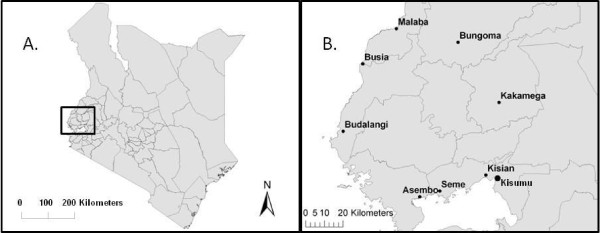
**Map of study area**. A. Map of Kenya showing where studies were conducted in western Kenya, indicated by the box. B. Map showing sampling locations in western Kenya study area, near Kisumu, Kenya's third largest city.

Adult and larval *A. gambiae *s.l. were sampled in the communities of Asembo from 1996 to 2010 and Seme from 2000 to 2008. These adjacent communities are situated 40-50 km west of the city of Kisumu along the northern shore of the Winam Gulf of Lake Victoria (Figure 1B), and are described in detail elsewhere [21,30,31]. The malaria vector populations in Asembo have been broadly exposed to pyrethroid insecticides in ITNs for about 13 years, beginning with Asembo's involvement in a village randomized, controlled trial of permethrin-treated bed nets in 1997. Initially, half of the villages in Asembo were provided permethrin-treated bed nets. Nets were re-treated with permethrin at approximately six-month intervals [23]. From 1999-2003, all villages were provided permethrin-treated bed nets and free house-to-house re-treatment [30]. Beginning in 2003, permethrin was replaced with alphacypermethrin (40 mg/m^2^) at 9 to 11 month intervals until the end of 2006. In early 2007, nets were treated with a KO Tab 1-2-3 wash resistant retreatment kit containing deltamethrin at a dose of 25 mg/m^2 ^[32]. Thereafter, residents were responsible for insecticide retreatment of their nets.

At the conclusion of the large-scale trial in Asembo in 2000, entomological studies began in Seme, located on Asembo's eastern border [21,31]. Seme did not participate in or receive ITNs as part of the cluster randomized trial that was conducted in Asembo. Net ownership in Seme began to rise only after the Kenya Ministry of Health and other partners increased access to ITNs through free and subsidized distributions beginning in 2004. ITNs were initially available to pregnant women and children <5 years old at a subsidized price at government-run health clinics. In 2006, ITNs were distributed free to children <5 years of age as part of a mass campaign [33] and the subsidy for ITNs distributed at government clinics was significantly increased. By 2009, ITNs were distributed at clinics to pregnant women and children <1 year of age free of charge.

Mosquito sampling methods have been described previously [21]. Sampling commenced in 1996 when adult female *A. gambiae *s.l. mosquitoes were collected from bed-net traps. From 1997 to 1999, mosquitoes were collected using pyrethrum spray catches (PSCs) inside houses in Asembo using 0.025% pyrethrum and 0.1% piperonyl butoxide mixed in kerosene. Houses for sampling were identified by selection from a geographic information system database [21,31,34]. From 2003-2008, mosquitoes were collected along transects running east to west during and immediately after the rainy season of each year (typically, April to July). From 2007 to 2010, adult samples were supplemented with larvae sampled from aquatic habitats along the same transect [21]. All larva-positive habitats encountered were exhaustively sampled for L3 and L4 stage larvae to obtain specimens for genotyping and bioassays as needed. Larvae and adults were morphologically identified to the *A. gambiae *complex, and categorized by stage; adults were categorized by sex, and by abdominal status if an adult female. Mosquitoes were desiccated in anhydrous calcium sulfate or silica gel for 48 hours. Following desiccation, samples were stored at room temperature until DNA extraction (see below).

### Geographic variation in *kdr *allele

In 2009 and 2010, the geographic scope of the study was expanded to estimate the frequency of the *kdr *L1014S allele in *A. gambiae *and *A. arabiensis *regionally in western Kenya to include Budalangi (2010 only), Kisian, Kakamega, Bungoma (2009 only), Malaba, and Busia in Nyanza and Western provinces (Figure 1A, B). Adult and larval stages of *A. gambiae *s.l. were sampled and processed from these other sites using similar procedures as for Asembo and Seme.

### Species identification and genotyping

DNA was extracted from *A. gambiae *s.l. larvae and adults following the protocol of Collins *et al *[35]. For adult males and larvae, whole bodies were ground in the initial step. For fed and half-gravid females, DNA was extracted from legs and wings only, while extraction from unfed and gravid females utilized legs, wings, and abdomens. Conventional polymerase chain reaction (PCR) was used to distinguish between the two sibling species of the *A. gambiae *species complex native to western Kenya, *A. gambiae *and *A. arabiensis *[36]. Real-time polymerase chain reaction (RT-PCR) was used to determine *kdr *genotype at amino acid position 1014 of the voltage-gated sodium channel. Following a modified version of the protocol by Bass et al. [37] , samples were genotyped using probes for the wild type (5'-CTTACGACTAAATTTC-3', labeled with HEX) and L1014S (5'-ACGACTGAATTTC-3', labeled with 6-FAM) alleles. A subset of samples was also genotyped for the L1014F allele (5'-ACGACAAAATTTC-3', labeled with 6-FAM). RT-PCR reactions were run on a Stratagene MxPro 3000 machine using a 96-well format. Each reaction included 5.0 μl of 2× Taqman RT-PCR master mix (Applied Biosystems), 0.2 μM *kdr *forward primer (5'-GCTGCGAGTTGTAGAGATGCG-3'), 0.2 μM *kdr *reverse primer (5'-GCTTACTGGTTTGGTCGGCATGT-3'), the wild type and L1014S probes at respective concentrations of 0.2 μM and 0.15 μM, ~50 ng DNA template, and sterile water in a final volume of 10 μl. Each 96-well plate included positive controls for all three genotypes in triplicate along with a no-template negative control. PCR conditions included an initial melting step at 95°C for 10 minutes followed by 45 cycles of 95° for 25 seconds and 64°C for 1 minute. Reaction curves for each set of reactions were visualized using Stratagene MxPro QPCR software and genotypes were scored by eye. Absence of alleles was confirmed by the text reports generated by the Stratagene MxPro QPCR software.

### Sporozoite infection association with *kdr*

To test the hypothesis that mosquitoes with the *kdr *L1014S allele are longer lived, more fit, and therefore more likely to acquire a malaria infection in the context of high ITN coverage, a case-control study was conducted using specimens collected in Asembo and Gem, an area just to the north of Asembo and to the northwest of Seme, from 2003 through 2007. Mosquitoes were collected monthly using light traps set overnight next to persons sleeping under bed nets. Female *A. gambiae *s.l. were tested for the presence of *Plasmodium falciparum *sporozoites using the antigen-capture ELISA method of Wirtz *et al *[38]. All ELISA positive *A. gambiae *s.l. were identified to species by PCR, and each *A. gambiae *was analysed for the L1014S allele as described above. For each ELISA positive *A. gambiae*, three ELISA-negative *A. gambiae *mosquitoes collected in the same village in the same month were randomly selected as controls for a matched analysis, and were genotyped for the *kdr *allele as well.

### Bioassays

To assess insecticide resistance phenotypically, field-collected *A. gambiae *s.l. mosquitoes were assayed for susceptibility to various insecticides using WHO tube tests [39]. Mosquitoes were collected from study sites as larvae and transported to an insectary, where they were reared in spring water and fed daily a mixture of finely ground fish food and brewer's yeast. Upon pupation, individuals were transferred to cages (40 cm^3 ^metal frames covered with untreated mosquito netting) and allowed to emerge. Upon emergence, all adults were held for 2-5 days and provided sugar solution until used in bioassays. Insecticide impregnated papers for five insecticides were obtained from WHO at standard concentrations for determining resistance in field populations [39]: bendiocarb (0.1%), DDT (4%), deltamethrin (0.05%), malathion (5%), and permethrin (0.75%). Bioassays performed in early 2009 only used DDT, deltamethrin, and permethrin, while those performed in late 2009 and 2010 used all five insecticides. Due to variation in larval numbers, as well as time to pupation and emergence, the number of replicates per insecticide and number of mosquitoes per assay varied. The number of individuals used in any single assay ranged from 8 - 20 and the minimum number of replicates was three. For each bioassay, the plastic tube from the test kit was lined with the appropriate test paper and mosquitoes were transferred from a holding cage to the tube via an aspirator. Following transfer, tubes were placed horizontally on a flat surface to ensure maximal contact with the impregnated papers. Exposure for each replicate was 60 minutes for all insecticides, after which individuals were transferred to cohort cages (approximately 1 L volume) and allowed to recover. During recovery, mosquitoes were provided with sugar solution and maintained at 25˚C and relative humidity ≥ 80%. Mortality was scored at 24 hours post-bioassay. Following the late 2009 and 2010 bioassays, DNA was extracted from assayed individuals and used to determine *kdr *L1014S genotype and species identification as described above.

### Data analysis

For analysis of changes in the frequency of the *kdr *L1014S allele over time, samples collected from each year and location were treated as separate populations. The frequency of L1014S homozygotes was analysed using multiple logistic regression in Stata (v. 11.0) with the dependent variable being the log_e_(odds of *kdr *L1014S homozygosity), modeled as a function of year and location. The frequency of *kdr *L1014S homozygotes, rather than frequency of *kdr *L1014S alleles, was selected as the dependent variable because the *kdr *mutation is only partially recessive and functionally more significant in the homozygous state [13,16]. For the 2009 and 2010 samples, the frequency of *kdr *L1014S homozygotes was modeled in a similar manner but with sub-population and month of collection as the independent variables.

Similarly, for analysis of spatial variability, samples collected in 2009-10 were analysed separately by location, as were samples from the same location collected at different times of the year. However, because previous population-genetic studies of *A. gambiae *suggest that western Kenya has a single, panmictic population [40], the 2009 and 2010 location-specific samples are referred to as sub-populations throughout to acknowledge their genetic non-independence. For each sub-population, genotype frequencies were compared to Hardy-Weinberg expectations using the program Genepop (v. 4.0), Option 1 (Hardy-Weinberg Exact Tests), Sub-option 3 (Probability test) [41]. Genepop was also used to estimate Wright's inbreeding coefficient (F_IS_) using the method of Weir and Cockerham [42]; and for sub-populations out of Hardy-Weinberg equilibrium, these values were used to test for heterozygote deficiency and excess (Genepop Option 1, Sub-options 1 and 2, respectively) using the U test as described in Raymond and Rousset [41]. A Bonferroni correction was applied to the level of significance for each procedure to adjust for multiple tests. To determine if mosquitoes with the *kdr *genotype were more likely to be infected with *P. falciparum *sporozoites, PROC LOGISTIC in SAS was used to model sporozoite infection status against *kdr *genotype.

Data from bioassays were pooled for each sub-population and insecticide, and mortality was calculated as the percentage of individuals that died within 24 hours of exposure. For bioassays performed in 2009, variation in survival was assessed using multiple logistic regression with the dependent variable being the log_e_(odds of survival) modelled as a function of sub-population and insecticide. Following regression, estimates of binomial 95% percent confidence intervals were used to compare bioassay outcomes between insecticides within a given sub-population. Further, levels of resistance were classified according to WHO guidelines [39]. Those with an overall mortality ≥ 98% were considered susceptible, those with mortality <98% but ≥ 80% were considered potentially resistant, and those with mortality <80% were strongly suspected to be resistant.

## Results

### Temporal variation in *kdr *genotype frequencies and species composition

**Figure 2:**
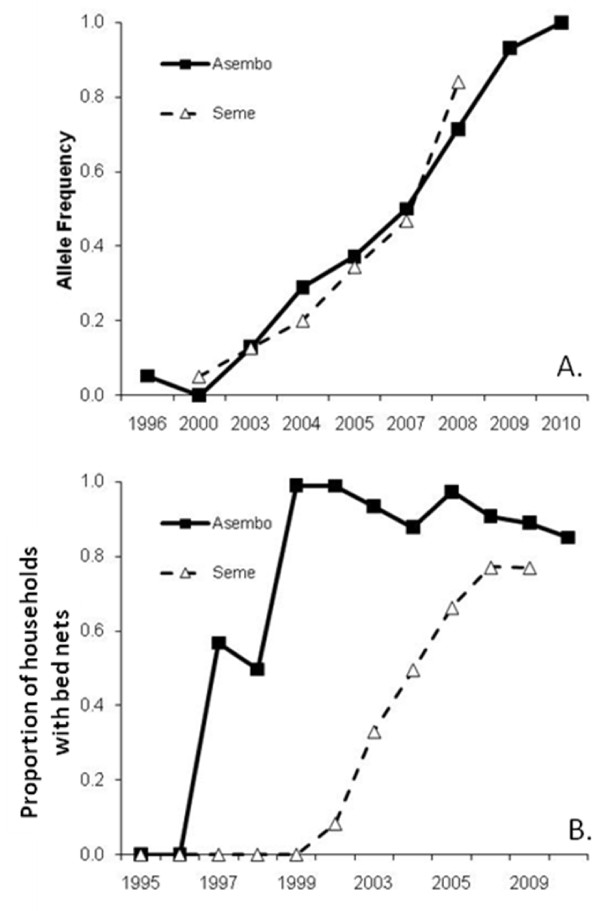
**Frequency of the *kdr *L1014S allele in *Anopheles gambiae *s.s. (top) and bed net ownership (bottom) by year in Asembo and Seme, western Kenya**. Bed net ownership is the percentage of households that own at least one bed net, including both treated conventional nets and long-lasting impregnated nets.

A total of 799 samples of *A. gambiae *were genotyped for the *kdr *L1014S mutation in sub-populations from Asembo (1996-2010) and Seme (2000-2008). Results showed a sharp increase in homozygote frequencies from complete absence in both locations initially to 80.5% for Seme in 2008 and 91.7% for Asembo in 2010 (Table 1, Figure 2). Logistic regression indicated that the proportion of *kdr *L1014S homozygotes increased significantly by year for both Asembo (O.R. = 2.108, 95% CI = 1.81 - 2.45, P < 0.001) and Seme (2.547, 95% CI = 2.04 - 3.17, P < 0.001). Exact tests for Hardy-Weinberg equilibrium for each year and location showed that genotype frequencies differed significantly from expectation only in 2008 after correcting for multiple tests (Table 1). The West African allele (1014F) was not detected in any genotyped *A. gambiae *individuals (n = 88) from Asembo and Seme in 2008. A single *A. arabiensis *from Busia that was homozygous for the *kdr *1014S allele was detected in 44 *A. arabiensis *from Bungoma, Busia, Kakamega, and Kisian in 2009 and 2010.

**Table 1:** Genotype frequencies for *kdr *1014S in *Anopheles gambiae *s.s. according location and date of sample collection.

Location	**Year/Month**^**a**^	Sample Size	**Genotype Counts**^**b **^**S/S S/E E/E**			*kdr *Homo- zygosity	S.E.	**F**_**IS**_^**c**^
Asembo	1996	95	85	10	0	0.0	--	-0.0503
	2000	27	27	0	0	0.0	--	--
	2004	126	69	41	16†	12.7	2.98	0.2053*
	2005	98	42	39	17	17.4	3.94	0.1537
	2007	20	7	6	7	35.0	10.94	0.4213
	2008	56	13	6	37‡	66.1	6.38	0.7416***
	2009	36	2	1	33†	91.7	4.67	0.7904**
	2010	8	0	0	8	100	0.00	--
Seme	2000	30	27	3	0	0.0	--	-0.0357
	2003	130	102	23	5†	3.8	1.69	0.1934*
	2005	116	50	52	14	12.1	3.04	0.0122
	2007	16	6	5	5	31.2	11.97	0.4000
	2008	41	5	3	33‡	80.5	6.27	0.7315***
Busia	2009/Apr	129	11	15	103‡	79.8	3.55	0.5295***
	2009/May	107	0	5	102	95.3	2.05	-0.0192
	2009/Jul	76	1	1	74†	97.4	1.85	0.6637*
	2010/May	134	0	3	131	97.8	1.28	-0.0076
Malaba	2009/May	378	2	25	351	92.9	1.33	0.1049
	2009/Jul	83	0	3	80	96.4	2.06	-0.0123
Bungoma	2009/May	38	0	1	37	97.4	2.63	--
	2009/Oct	100	3	3	94‡	94.0	2.39	0.6538***
	2009/Nov	36	1	2	33	91.7	4.67	0.4815
	2010/May	152	4	1	147	96.7	1.45	0.8862***
Kakamega	2009/Apr	67	1	2	64†	95.5	2.55	0.4903*
	2009/May	22	1	1	20	90.9	6.27	0.6557
	2009/Jul	39	0	1	38	97.4	2.56	--
	2009/Sep	46	2	0	44‡	95.6	3.04	1.0000***
	2010/May	38	0	1	37	97.4	2.63	--
Kisian	2009/Feb	27	0	1	26	96.3	3.70	--
	2009/Jun	27	1	1	25†	92.6	5.14	0.6579*
	2010/May	33	0	0	33	100.0	0.00	--

^a^Year of sample collection only is given for the *kdr *time series for Asembo and Seme although sampling was typically performed during the peak of the rainy season in May or June. Month of sampling is given for the 2009 and 2010 collections from sites across western Kenya.

^b^Three possible *kdr *genotypes are S/S, S/E, and EE, where S represents the susceptible wild type allele and E (for East African) represents the L1014S allele. The symbols † and ‡ indicate that genotype frequencies depart from Hardy-Weinberg expectations, although those with † are no longer significant following a Bonferroni correction for multiple tests

^c^F_IS _values > 0 indicate heterozygote deficiency, while values < 0 indicate heterozygote excess. A double dash (--) indicates values that were undefined. Significance level indicates rejection of the null hypothesis F_IS _= 0, where *, **, and *** indicate p < 0.05, p < 0.01, and p < 0.001, respectively. After a Bonferroni correction for multiple tests, only those values with *** remain significant at the corrected significance level (α = 0.0017).

### Geographic variation in *kdr *genotype frequencies and species composition

**Figure 3:**
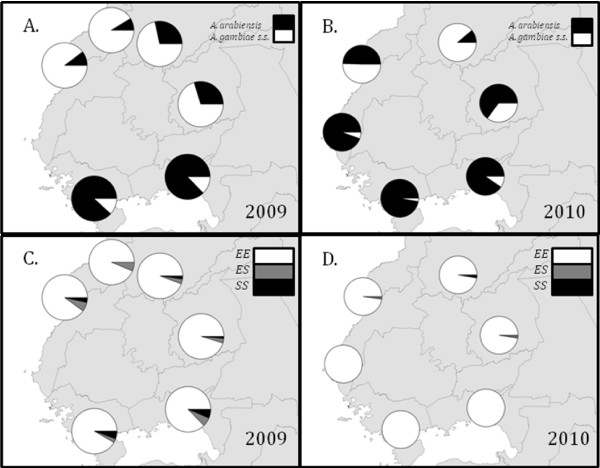
Proportion of *A. gambiae *s.s. and *A. arabiensis *at sites in western Kenya in 2009 (A) and 2010 (B); Proportion of *kdr *genotypes observed in *A. gambiae *s.s. at sites in western Kenya in 2009 (C) and 2010 (D).

3,172 specimens were identified to species within the *A. gambiae *complex from seven sites from 2009 and 2010. The proportion of the sibling species *A. gambiae *and *A. arabiensis *varied among sampling location but patterns were similar between 2009 and 2010 (Table 2, Figure 3A, B). In 2009, the percentage of *A. gambiae *was highest in Busia (89.7%) and Malaba (91.2%), close to the border with Uganda, and lowest at sites to the southeast near Lake Victoria, namely Kisian (12.8%) and Asembo/Seme (11.8%). In 2010, the percentage of *A. gambiae *were similarly lowest at Kisian (9.5%) and Asembo/Seme (2.8%), but were highest in Bungoma (88.9%) rather than Busia (50.4%). Overall, the percentage of *A. arabiensis *in all samples was higher in 2010 compared to 2009.

**Table 2:** Species composition in combined samples of *Anopheles gambiae *s.l. from sites in western Kenya collected in 2009 and 2010.

Location	Year	***Anopheles gambiae *s.s**.	*Anopheles arabiensis*	Total Genotyped
Busia	2009	89.7 (347)	10.3 (40)	387
	2010	50.4 (134)	49.6 (132)	266
Malaba	2009	91.2 (479)	8.8 (46)	525
	2010	-	-	-
Bungoma	2009	71.6 (78)	28.4 (31)	109
	2010	88.9 (152)	11.1 (19)	171
Kakamega	2009	70.3 (211)	29.7 (89)	300
	2010	35.2 (38)	64.8 (70)	108
Asembo/Seme	2009	11.8 (38)	88.2 (284)	322
	2010	2.8 (4)	97.2 (141)	145
Kisian	2009	12.8 (63)	87.2 (430)	493
	2010	9.5 (33)	90.5 (313)	346

In each column, the proportion of each species is presented by location and year with the absolute number of each species presented in parentheses. The total number of *A. gambiae *s.l. that were genotyped is provided in the last column.

A total of 1,532 *A. gambiae *were genotyped for the *kdr *L1014S allele from samples taken in 2009 and 2010 in Busia, Malaba, Bungoma, Kakamega, and Kisian (Table 1). The frequency of *kdr *L1014S homozygotes in *A. gambiae *was high at all sites, and did not differ by location for either year (P = 0.254) (Table 1, Figure 3C, D). Within locations, homozygote frequency only differed between collections for one site, Busia in 2009 (O.R. = 2.67, 95% CI = 1.51 - 4.73, P < 0.01, Table 1). When including this site in the analysis, all locations had *kdr *L1014S homozygote frequencies of 79% or greater; but when excluding the Busia sample from April of 2009, homozygote frequencies were all greater than 90%. There was no statistical difference in genotype frequencies between larval and adult samples (O.R. = 1.15, 95% CI = 0.73 - 1.84, P = 0.537). Estimates of Wright's inbreeding coefficient (F_IS_) for both the time-series and 2009/2010 sub-populations indicated that genotype frequencies out of Hardy-Weinberg equilibrium tended toward heterozygote deficiency (Table 1).

### Sporozoite infection

Between 2003 and 2007, a total of 43 *A. gambiae *mosquitoes were found to be infected with *P. falciparum *by sporozoite ELISA. These were genotyped for *kdr *L1014S along with 129 control mosquitoes. Among the sporozoite positive mosquitoes, 11.6% were homozygous for the *kdr *L1014S allele. Among sporozoite negative mosquitoes, 5.4% were homozygous for the *kdr *L1014S allele (Table 3). However, these percentages were not significantly different (P = 0.177).

**Table 3:** *kdr *L1014S genotype frequencies of *A. gambiae *s.s. from 2003-2007 in association with sporozoite infection.

	*kdr *L1014S genotype			
ELISA	SS	ES	EE	Total
Negative	64 (49.6)	58 (45.0)	7 (5.4)	129 (100)
Positive	21 (48.8)	17 (39.5)	5 (11.6)	43 (100)

### Bioassays

A total of 3,432 individual *A. gambiae *s.l. were tested in phenotypic assays in 2009, and 1,027 individuals in 2010. Samples varied in their phenotypic susceptibility to different insecticides in standardized WHO bioassays. Of the three compounds tested in 2009, mosquitoes (grouped only within species complex) were generally most susceptible to deltamethrin, least susceptible to DDT, and intermediate in susceptibility to permethrin (Table 4). However, in late 2009/2010, there was more variability in the response to different insecticides with resistance highest to deltamethrin in *A. gambiae *from Bungoma. In Busia, the highest degree of resistance was to permethrin. Susceptibility to malathion was nearly 100% in all populations. There were marked differences among species, with *A. arabiensis *being susceptible to all insecticides, and *A. gambiae *having reduced susceptibility to most insecticides.

**Table 4:** Percent mortality in WHO bioassays 24 hours after exposure to discriminating concentrations of insecticide.

Species	Sub-Population	Year	DDT	Permethrin	Deltamethrin	Bendiocarb	Malathion
*A. gambiae *s.l.	Bungoma	2009	**78 (54)**	84 (148)	89 (88)	ND	ND
	Busia	2009	**21 (78)**	**54 (347)**	**78 (249)**	ND	ND
	Kakamega	2009	**78 (188)**	85 (225)	87 (342)	ND	ND
	Kisian	2009	ND	84 (231)	91 (22)	ND	ND
	Malaba	2009	**64 (59)**	**68 (627)**	89 (774)	ND	ND
							
*A. gambiae *s.s.	Bungoma	2009-2010	**62 (37)**	**74 (74)**	**42 (19)**	96 (25)	100 (44)
	Busia	2009-2010	**33 (15)**	**16 (25)**	**48 (21)**	**79 (19)**	100 (15)
							
*A. arabiensis*	Asembo	2010	100 (36)	97 (64)	83 (29)	100 (34)	100 (24)
	Budalangi	2010	100 (23)	100 (21)	100 (25)	94 (17)	100 (32)
	Busia	2009-2010	98 (42)	87 (23)	100 (18)	93 (15)	100 (18)
	Kakamega	2009-2010	100 (8)	82 (11)	100 (15)	82 (11)	100 (10)
	Kisian	2009-2010	100 (32)	87 (63)	94 (70)	100 (24)	100 (16)

The number of adult mosquitoes assayed (*n*) is given in parentheses and bioassays with mortality <80% are highlighted in boldface.

^a^Mortality not determined.

## Discussion

Three processes have marked the dynamics of the *Anopheles gambiae *species complex in western Kenya during the course of national scale-up of ITNs: (1) a decline in density of indoor resting mosquitoes [31,43], (2) a shift from a predominance of *A. gambiae *to *A. arabiensis *in adult and larval stages [21], and (3) a decrease in longevity of adult females, but no change in host preference patterns [21]. These changes correlate with a sustained reduction in the malaria burden in the human population [30]. The rapid rise in the frequency of the *kdr *L1014S allele to near fixation in *A. gambiae *as reported here, could herald a reversal of these positive outcomes. The marked rise in frequency of the *kdr *L1014S allele over 13 years in Asembo and Seme suggests that *A. gambiae *has been undergoing strong selection for resistance to the pyrethroid insecticides used in ITNs distributed there. Allele frequencies ranged from 2.5% to 3.8% in *A. gambiae *in western Kenya in 1987 [27]. The *kdr *allele frequency we report from Asembo for 1996 (5.3%, n = 95) is nearly identical to that of Stump *et al *[27] for the same year. Therefore, when ITNs were distributed in Asembo for the intervention trial in 1996, alleles for *kdr*-mediated resistance were already present in the *A. gambiae *population. Although direct comparisons with other populations are confounded by many factors, the rise in the L1014S allele observed in western Kenya was nearly as rapid as that observed for the L1014F allele in Ghana [16].

The rise in the mutation's frequency followed similar trajectories in Asembo and Seme despite substantial differences in net coverage between the areas for a decade (Figure 2). Net ownership rose modestly in Seme from 2000 to 2003 and then substantially increased with subsidized distribution of ITNs to pregnant women and children <5 years of age from government health facilities beginning in 2004, followed by a mass campaign in 2006 [21,33]. The similar patterns of emergence of resistance likely reflect details of migration and selection pressure not measured in our study. While *A. gambiae *came into ever increasing contact with pyrethroids in Asembo over the past 13 years, selection pressures outside of Asembo due to either malaria control or agricultural pesticide use are unknown.

Other studies have also reported increases in frequency of the *kdr *L1014S allele in *A. gambiae *from Burundi [44] and Uganda [45] associated with vector control using pyrethroid insecticides; while studies in Niger [46] and Equatorial Guinea [47] have observed sharp rises in the *kdr *L1014F allele in response to ITNs and IRS, respectively. In Burundi and Rwanda, the rise in the *kdr *L1014S allele was ascribed to use of insecticides as indoor residual sprays to control adult stages of malaria vectors. However, in Burundi, the allele frequency actually increased in an unsprayed control area as well as in the sprayed area. In Uganda, there were marked regional variations in allele frequency without clear correlation with intensity of use of IRS or ITNs, and allele frequency and phenotypic resistance were noted particularly in areas with a history of cotton agriculture where insecticide use is often intense [45].

In other settings, agricultural use of insecticides has been cited as the primary cause of the emergence of insecticide resistance in *A. gambiae *s.l. populations in sub-Saharan Africa [48-50]. *Anopheles gambiae *s.l. larvae may face strong selection pressure for resistance to insecticides if exposed in breeding sites located near cultivated fields where insecticides are applied to control agricultural pests. In a cotton-growing region of northern Cameroon, investigators sampled mosquito larvae from breeding sites within cotton fields at different times during the rainy season [51]. Bioassays of emerged adults revealed that vector susceptibility to DDT and permethrin decreased over time in accordance with a spraying schedule in the cotton fields that included two applications of an organochlorine compound (endosulfan) followed by a pyrethroid/organophosphate mixture (cypermethrin/profenofos). In Burkina Faso, the spatial heterogeneity of *kdr *L1014F was associated with cotton agriculture; the mutation was present at 16 of 21 sites and ranged in frequency from 4.7% to 97.0% with the highest frequencies occurring in the so-called "cotton belt" [52]. Although data are lacking, current agricultural use of pesticides in western Kenya is likely low owing to the prevalence of subsistence agriculture. However, use of permethrin for cattle dips is common, and the region produces some cash crops (sugar cane and cotton) that may require insecticide use. In addition, the low level of resistance to bendiocarb implies that agricultural use of insecticides may play a role in the evolution of resistance in malaria vectors in western Kenya. However, the strong temporal association reported here between net ownership and *kdr *L1014S frequency (Figure 2) suggests that ITNs have been the most important selection pressure in this study population.

Given the trends in net ownership and *kdr *L1014S frequency in Seme, it is not surprising that similarly high homozygote frequencies were found in samples of *A. gambiae *throughout western Kenya in 2009 and 2010. One unexpected finding is the apparently greater magnitude of phenotypic resistance near the Ugandan border, as well as the dramatic differences in species composition near Lake Victoria compared to sites further north. Due to the decline in *A. gambiae *along the lakeshore, inadequate numbers of this species were available for testing in phenotypic assays. It is, therefore, possible that phenotypic resistance in *A. gambiae *along the lakeshore is similar to that of sites further away and the few mosquitoes that were tested (<5 per insecticide) suggest that this is the case. Therefore, the question is why *A. gambiae *remains the predominant species in sites located further from the lakeshore. The current hypothesis is that ITNs have not been in place sufficiently long in Busia, Malaba, Bungoma, and Kakamega to drive down local abundance of *A. gambiae*. Qualitative comparisons of the ratio of *A. arabiensis *to *A. gambiae *amongst all these sites suggests that the former species is rising in frequency in the sites away from the lake shore as it has at sites near the lake [21]. Indeed, the presence of *A. arabiensis *at Bungoma and Kakamega, both sites at relatively high altitudes where *A. gambiae *has traditionally been the only species in the complex present, is particularly striking [53]. However, because historical data on net ownership in these areas are lacking, baseline levels prior to the national scale-up of ITN are unknown. In Asembo and Seme, the change from sub-populations dominated by *A. gambiae *to those dominated by *A. arabiensis *took about a decade and occurred in Asembo first, as would be expected if ITNs were the primary cause [21]. This hypothesis is one that could be tested by monitoring both ITN coverage and species composition over the next several years in the region.

One trend that was consistent throughout all populations examined was the high degree of susceptibility of *A. arabiensis *to all insecticides but moderate to high resistance to pyrethroids in *A. gambiae*. The persistence of a species with little to no pyrethroid resistance (*A. arabiensis*) compared to a species with moderate to high levels of pyrethroid resistance (*A. gambiae*) in an area with high ITN coverage is somewhat counterintuitive. However, it is likely explained by the behaviour of *A. arabiensis *which often feeds outdoors and on cattle and may avoid the insecticide on nets. *Anopheles arabiensis *populations are therefore able to persist, apparently with little to no selection from the pyrethroid insecticides on nets [21]. *Anopheles gambiae *populations, despite having some resistance to pyrethroid insecticides, are still in decline possibly due to irritancy of the insecticide on the nets or the physical barrier imposed by the nets. These observations also suggest there is a limit to the degree of resistance conferred by the molecular and biochemical mechanisms currently present in western Kenya. Similar observations were made in the early 1990s during a small scale study of permethrin-treated nets where resistance was detected within a year of implementation [24], but reached a plateau and even regressed after three years [25]. However, other resistance mechanisms, possibly coupled with secondary compensatory mutations, may lead to further increases in pyrethroid resistance which could lead to a resurgence of *A. gambiae*. Despite the rapid decline in *A. gambiae *along the lakeshore, malaria transmission-presumably maintained by *A. arabiensis*--remains high with parasite prevalence in children over 45% (M. Hamel, unpublished data). As ITNs and IRS are increasingly scaled up throughout Africa, behavioural avoidance of these interventions may become increasingly important and tools to address species or populations exhibiting these traits are urgently needed.

Although pyrethroid resistance has been reported locally or regionally in many parts of sub-Saharan Africa, the impact of resistance on vector control is not always consistent between locations. In West Africa, for example, ITNs treated with the pyrethroid lambdacyhalothrin remained effective in reducing malaria prevalence in the face of *kdr *L1014F resistance in Cote d'Ivoire [54]. In contrast, the failure of IRS using pyrethroids in Bioko Island was associated with a high frequency of the *kdr *L1014F allele [47], while in Benin, N'Guessan *et al *[55] reported significantly reduced effectiveness of both ITNs and IRS in a region where *kdr *1014F frequency was 83%. Data from western Kenya suggest that the rise of the *kdr *allele has had limited impact on the effectiveness of ITNs at least at sites along the lakeshore. Annual malaria surveys in Asembo indicated a decline in the prevalence of malaria until 2008. However, prevalence rose in 2009 and remained high in 2010 (M. Hamel, unpublished data). While the rise in malaria coincided with the period when the *kdr *allele was peaking in *A. gambiae*, entomologic data suggest that increasing pyrethroid resistance in this species is not the reason for increasing malaria in Asembo. The shift from a population dominated by *A. gambiae *to *A. arabiensis *along with the analysis of sporozoite rates by *kdr *genotype indicates that the rise of the *kdr *L1014S allele has not compromised the efficacy of ITNs along the Lake Victoria basin. On the other hand, the persistence of *A. gambiae *in sites further from the lakeshore where detectable levels of phenotypic resistance were observed is more worrisome. Nevertheless, the decline in *A. gambiae *relative to *A. arabiensis *from 2009 to 2010 in these sites where *A. arabiensis *has traditionally been rare or absent suggests that the hypothesis that these areas are more recent recipients of ITNs is correct and further increases in ITN coverage may continue to suppress *A. gambiae *populations to the levels observed along the lakeshore. However, the possibility that further increases in insecticide resistance, possibly attributable to changes in metabolic enzymes associated with pyrethroid resistance, are spreading east cannot be discounted. Continued surveillance of these populations is needed to monitor for additional changes in insecticide resistance and to assess its impact on the effectiveness of ITNs.

## Conclusions

While the continuing effectiveness of ITNs against *A. gambiae *populations along the Lake Victoria basin is encouraging, the dramatic rise of the *kdr *L1014S allele and the detection of phenotypic resistance to pyrethroid insecticides in this region is alarming. Control failure due to insecticide resistance can arise rapidly, as has been observed in IRS programs in South Africa [56] and Equatorial Guinea [47]. However, it is possible that low levels of insecticide resistance existed undetected in these areas and only became apparent when IRS was no longer effective. *Anopheles gambiae *populations in western Kenya may be primed for further increases in pyrethroid resistance to a point where ITNs begin to lose their effectiveness. Monitoring is, therefore, imperative to quantify changes in insecticide resistance and to elucidate the specific resistance mechanisms operating in the populations, so that mitigation strategies can be implemented if and when pyrethroid resistance compromises the effectiveness of ITNs.

## Competing interests

The authors declare that they have no competing interests.

## Authors' contributions

DKM, MNB, JMV, MJH, WAH, EDW and JEG designed the study and wrote the manuscript. DKM, EO, DM, LK performed species identification and RT-PCR analysis of *A. gambiae *kdr genotypes. EO, FA, MO and GO conducted and analysed the phenotypic bioassay data. DKM, EO, EDW and JEG analysed the data. All authors read and approved the final manuscript.

## References

[B1] Becker-Dreps SI Biddle AK Pettifor A Musuamba G Imbie DN Meshnick S Behets F Cost-effectiveness of adding bed net distribution for malaria prevention to antenatal services in Kinshasa, Democratic Republic of the Congo Am J Trop Med Hyg 2009 81 496 502 19706921

[B2] Mueller DH Wiseman V Bakusa D Morgah K Dare A Tchamdja P Cost-effectiveness analysis of insecticide-treated net distribution as part of the Togo integrated child health campaign Malar J 2008 7 73 10.1186/1475-2875-7-73 PMC2396647 18445255

[B3] Yukich JO Zerom M Ghebremeskel T Tediosi F Lengeler C Costs and cost-effectiveness of vector control in Eritrea using insecticide-treated bed nets Malar J 2009 8 51 10.1186/1475-2875-8-51 PMC2670315 19331664

[B4] Lengeler C Insecticide-treated bednets and curtains for preventing malaria Cochrane Database Syst Rev 2004 2004 CD000363 10.1002/14651858.CD000363 10796535

[B5] W.H.O. World Malaria Report, 2010 2010 Geneva, Switzerland

[B6] Feachem RG Phillips AA Malaria: 2 years in the fast lane Lancet 2009 373 1409 1411 10.1016/S0140-6736(09)60801-1 19394520

[B7] Greenwood BM Control to elimination: Implications for malaria research Trends Parasitol 2008 24 449 454 10.1016/j.pt.2008.07.002 18760671

[B8] W.H.O. 6 Pesticides and their application for the control of vectors and pests of public health importance Department of Control of Neglected Tropical Diseases, WHO Pesticide Evaluation Scheme 2006 Geneva, Switzerland: World Health Organization

[B9] Hemingway J Hawkes NJ McCarroll L Ranson H The molecular basis of insecticide resistance in mosquitoes Insect Biochem Mol Biol 2004 34 653 665 10.1016/j.ibmb.2004.03.018 15242706

[B10] Li X Schuler MA Berenbaum MR Molecular mechanisms of metabolic resistance to synthetic and natural xenobiotics Annu Rev Entomol 2007 52 231 253 10.1146/annurev.ento.51.110104.151104 16925478

[B11] Soderlund DM Knipple DC The molecular biology of knockdown resistance to pyrethroid insecticides Insect Biochem Mol Biol 2003 33 563 577 10.1016/s0965-1748(03)00023-7 12770575

[B12] Martinez-Torres D Chandre F Williamson MS Darriet F Berge JB Devonshire AL Molecular characterization of pyrethroid knockdown resistance (*kdr*) in the major malaria vector *Anopheles gambiae *s.s Insect Mol Biol 1998 7 179 184 10.1046/j.1365-2583.1998.72062.x 9535162

[B13] Ranson H Jensen B Vulule JM Wang X Hemingway J Collins FH Identification of a point mutation in the voltage-gated sodium channel gene of Kenyan *Anopheles gambiae *associated with resistance to DDT and pyrethroids Insect Mol Biol 2000 9 491 497 10.1046/j.1365-2583.2000.00209.x 11029667

[B14] Pinto J Lynd A Vicente JL Santolamazza F Randle NP Gentile G Moreno M Simard F Charlwood JD do Rosário VE Caccone A Della Torre A Donnelly MJ Multiple origins of knockdown resistance mutations in the afrotropical mosquito vector *Anopheles gambiae* PLoS One 2007 2 e1243 10.1371/journal.pone.0001243 PMC2080755 18043750

[B15] Santolamazza F Calzetta M Etang J Barrese E Dia I Caccone A Donnelly MJ Petrarca V Simard F Pinto J della Torre A Distribution of knock-down resistance mutations in *Anopheles gambiae *molecular forms in west and west-central Africa Malar J 2008 7 74 10.1186/1475-2875-7-74 PMC2405802 18445265

[B16] Lynd A Weetman D Barbosa S Egyir-Yawson A Mitchell S Pinto J Hastings I Donnelly MJ Field, genetic and modeling approaches show strong positive selection acting upon an insecticide resistance mutation in *Anopheles gambiae *s.s Mol Biol Evol 2010 27 1117 1125 10.1093/molbev/msq002 PMC2877529 20056691

[B17] Busvine JR Experiments concerned with the development of World Health Organization test for resistance in adult mosquitoes Indian J Malariol 1958 12 279 286 13806453

[B18] Lockwood JA Sparks TC Story RN Evolution of insect resistance to insecticides: A reevaluation of the roles of physiology and behavior Bull Entomol Soc Am 1984 30 41 51

[B19] Roberts DR Andre RG Insecticide resistant issues in vectors Am J Trop Med Hyg 1994 50 Suppl 21 34 10.4269/ajtmh.1994.50.21 8024082

[B20] Siegert PY Walker ED Miller JR Differential behavioral responses of *Anopheles gambiae *(Diptera: Culicidae) modulate mortality caused by pyrethroid-treated bed nets J Econ Entomol 2009 102 2061 2071 10.1603/029.102.0607 20069832

[B21] Bayoh MN Mathias DK Odiere MR Mutuku FM Kamau L Gimnig JE Vulule JM Hawley WA Hamel MJ Walker ED *Anopheles gambiae*: historical population decline associated with regional distribution of insecticide-treated bed nets in western Nyanza Province, Kenya Malar J 2010 9 62 10.1186/1475-2875-9-62 PMC2838909 20187956

[B22] Beach RF Ruebush TK Sexton JD Hightower A Roberts J Evaluation of permethrin-impregnated bed-nets and curtains during seasonal periods of intense malaria transmission in western Kenya Am J Trop Med Hyg 1993 49 230 300 10.4269/ajtmh.1993.49.290 8372952

[B23] Phillips-Howard PA ter Kuile FO Nahlen BL Alaii JA Gimnig JE Kolczak MS Terlouw DJ Kariuki SK Shi YP Kachur SP Hightower AW Vulule JM Hawley WA The efficacy of permethrin-treated bednets on child mortality and morbidity in western Kenya. II: Study design and methods Am J Trop Med Hyg 2003 68 10 15 12749480

[B24] Vulule JM Beach RF Atieli FK Roberts JM Mount DL Mwangi RW Reduced susceptibility of *Anopheles gambiae *to permethrin associated with the use of permethrin-impregnated bednets and curtains in Kenya Med Vet Entomol 1994 8 71 75 10.1111/j.1365-2915.1994.tb00389.x 8161849

[B25] Vulule JM Beach RF Atieli FK Mount DL Roberts JM Mwangi RW Long-term use of permethrin impregnated nets does not increase *Anopheles gambiae *permethrin tolerance Med Vet Entomol 1996 10 71 79 10.1111/j.1365-2915.1996.tb00084.x 8834745

[B26] Vulule JM Beach RF Atieli FK McAllister JC Brogdon WG Roberts JM Mwangi RW Hawley WA Elevated oxidase and esterase levels associated with permethrin tolerance in *Anopheles gambiae *from Kenyan villages using permethrin-impregnated nets Med Vet Entomol 1999 13 239 244 10.1046/j.1365-2915.1999.00177.x 10514048

[B27] Stump AD Atieli FK Vulule JM Besansky NJ Dynamics of the pyrethroid knockdown resistance allele in Western Kenya populations of *Anopheles gambiae *in response to insecticide-treated bed net trial Am J Trop Med Hyg 2004 70 591 596 15210997

[B28] Chen H Githeko AK Githure JI Mutunga J Zhou G Yan G Monooxygenase levels and knockdown resistance (*kdr*) allele frequencies in *Anopheles gambiae *and *Anopheles arabiensis *in Kenya J Med Entomol 2008 45 242 250 10.1603/0022-2585(2008)45[242:mlakrk]2.0.co;2 PMC3726191 18402140

[B29] Kamau L Agai D Matoke D Wachira L Gikandi G Vulule JM Status of insecticide susceptibility in *Anopheles gambiae *sensu lato and *Anopheles funestus *mosquitoes from western Kenya J Insect Sci 2008 8 11 10.1673/031.008.1101 PMC3061582 20345290

[B30] Lindblade KA Eisele TP Gimnig JE Alaii JA Odhiambo F ter Kuile FO Hawley WA Wannemuehler KA Phillips-Howard PA Rosen DH Nahlen BL Terlouw DJ Adazu K Vulule JM Slutsker L Sustainability of reductions in malaria transmission and infant mortality in western Kenya with use of insecticide-treated bednets: 4 to 6 years of follow-up JAMA 2004 291 2571 2580 10.1001/jama.291.21.2571 15173148

[B31] Lindblade KA Gimnig JE Kamau L Hawley WA Odhiambo F Olang G ter Kuile FO Vulule JM Slutsker L Impact of sustained use of insecticide-treated bednets on malaria vector species distribution and culicine mosquitoes J Med Entomol 2006 43 428 432 10.1603/0022-2585(2006)043[0428:iosuoi]2.0.co;2 16619629

[B32] Yates A N'Guessan R Kaur H Akogbéto M Rowland M Evaluation of KO-Tab 1-2-3: a wash-resistant 'dip-it-yourself' insecticide formulation for long-lasting treatment of mosquito nets Malar J 2005 4 52 10.1186/1475-2875-4-52 PMC1291394 16269088

[B33] Hightower A Kiptui R Manya A Wolkon A Vanden Eng JL Hamel M Noor A Sharif SK Buluma R Vulule J Laserson K Slutsker L Akhwale W Bed net ownership in Kenya: the impact of 3.4 million free bed nets Malar J 2010 9 183 10.1186/1475-2875-9-183 PMC2912322 20576145

[B34] Hightower AW Ombok M Otieno R Odhiambo R Oloo AJ Lal AA Nahlen BL Hawley WA A geographic information system applied to a malaria field study in western Kenya Am J Trop Med Hyg 1998 58 266 272 10.4269/ajtmh.1998.58.266 9546401

[B35] Collins FH Mendez MA Rasmussen MO Mehaffey PC Besansky NJ Finnerty V A ribosomal RNA gene probe differentiates member species of the *Anopheles gambiae *complex Am J Trop Med Hyg 1987 37 37 41 10.4269/ajtmh.1987.37.37 2886070

[B36] Scott JA Brogdon WG Collins FH Identification of single specimens of the *Anopheles gambiae *complex by the polymerase chain-reaction Am J Trop Med Hyg 1993 49 520 529 10.4269/ajtmh.1993.49.520 8214283

[B37] Bass C Nikou D Donnelly MJ Williamson MS Ranson H Ball A Vontas J Field LM Detection of knockdown resistance (*kdr*) mutations in *Anopheles gambiae*: A comparison of two new high-throughput assays with existing methods Malar J 2007 6 111 10.1186/1475-2875-6-111 PMC1971715 17697325

[B38] Wirtz RA Zavala F Charoenvit Y Campbell GH Burkot TR Schneider I Esser KM Beaudoin RL Andre RG Comparative testing of monoclonal antibodies against *Plasmodium falciparum *sporozoites for ELISA development Bull WHO 1987 65 39 45 PMC2490858 3555879

[B39] W.H.O. Test procedures for insecticide resistance monitoring in malaria vectors: bio-efficacy and persistence of insecticides on treated surfaces 1998 Geneva, Switzerland: World Health Organization

[B40] Lehmann T Besansky NJ Hawley WA Fahey TG Kamau L Collins FH Microgeographic structure of *Anopheles gambiae *in western Kenya based on mtDNA and microsatellite loci Mol Ecol 1997 6 243 253 10.1046/j.1365-294x.1997.00177.x 9076979

[B41] Raymond M Rousset F Genepop (version-1.2) - Population-genetics software for exact tests and ecumenicism J Hered 1995 86 248 249

[B42] Weir BS Cockerham CC Estimating F-statistics for the analysis of population structure Evolution 1984 38 1358 1370 10.1111/j.1558-5646.1984.tb05657.x 28563791

[B43] Gimnig JE Vulule JM Lo TQ Kamau L Kolczak MS Phillips-Howard PA Mathenge EM ter Kuile FO Nahlen BL Hightower AW Hawley WA Impact of permethrin-treated bednets on entomological indices in an area of intense year-round malaria transmission Am J Trop Med Hyg 2003 68 suppl 16 22 12749481

[B44] Protopopoff N Verhaeghen K Van Bortel W Roelants P Marcotty T Baza D D'Alessandro U Coosemans M A significant increase in *kdr *in *Anopheles gambiae *is associated with an intensive vector control intervention in Burundi highlands Trop Med Int Health 2008 13 1479 1487 10.1111/j.1365-3156.2008.02164.x 18983277

[B45] Verhaeghen K Van Bortel W Roelants P Okello PE Talisuna A Coosemans M Spatio-temporal patterns in *kdr *frequency in permethrin and DDT resistant *Anopheles gambiae *s.s. from Uganda Am J Trop Med Hyg 2010 82 566 573 10.4269/ajtmh.2010.08-0668 PMC2844549 20348500

[B46] Czeher C Labbo R Arzika I Duchemin J Evidence of increasing leu-phe knockdown resistance mutation in *Anopheles gambiae *from Niger following a nationwide long-lasting insecticide-treated nets implementation Malar J 2008 7 189 10.1186/1475-2875-7-189 PMC2562389 18817574

[B47] Sharp BL Ridl FC Govender D Kuklinski J Kleinschmidt I Malaria vector control by indoor residual insecticide spraying on the tropical island of Bioko, Equatorial Guinea Malar J 2007 6 52 10.1186/1475-2875-6-52 PMC1868751 17474975

[B48] Diabate A Baldet T Chandre F Akogbeto M Guiguemde TR Darriet F Brengues C Guillet P Hemingway J Small GJ Hougard JM The role of agricultural use of insecticides in resistance to pyrethroids in *Anopheles gambiae *s.l. in Burkina Faso Am J Trop Med Hyg 67 617 622 200 10.4269/ajtmh.2002.67.617 12518852

[B49] Lines JD Do agricultural insecticides select for insecticide resistance in mosquitoes? A look at the evidence Parasitol Today 1988 4 S17 S20 10.1016/0169-4758(88)90083-x 15463085

[B50] Müller P Chouaïbou M Pignatelli P Etang J Walker ED Donnelly MJ Simard F H R Pyrethroid tolerance is associated with elevated expression of antioxidants and agricultural practice in *Anopheles arabiensis *sampled from an area of cotton fields in Northern Cameroon Mol Ecol 2008 17 1145 1155 10.1111/j.1365-294X.2007.03617.x 18179425

[B51] Chouaibou M Etang J Brevault T Nwane P Hinzoumbe CK Mimpfoundi R Simard F Dynamics of insecticide resistance in the malaria vector *Anopheles gambiae *s.l. from an area of extensive cotton cultivation in northern Cameroon Trop Med Int Health 2008 13 476 486 10.1111/j.1365-3156.2008.02025.x 18248566

[B52] Dabire KR Diabate A Namountougou M Toe KH Ouari A Kengne P Bass C Baldet T Distribution of pyrethroid and DDT resistance and the L1014F *kdr *mutation in *Anopheles gambiae *s.l. from Burkina Faso (west Africa) Trans R Soc Trop Med Hyg 2009 103 1113 1120 10.1016/j.trstmh.2009.01.008 19246066

[B53] Shililu JI Maier WA Seitz HM Orago AS Seasonal density, sporozoite rates and entomological inoculation rates of *Anopheles gambiae *and *Anopheles funestus *in a high-altitude sugarcane growing zone in Western Kenya Trop Med Int Health 1998 3 706 710 10.1046/j.1365-3156.1998.00282.x 9754665

[B54] Henry MC Assi SB Rogier C Dossou-Yovo J Chandre F Guillet P Carnevale P Protective efficacy of lambda-cyhalothrin treated nets in *Anopheles gambiae *pyrethroid resistance areas of Cote d'Ivoire Am J Trop Med Hyg 2005 73 859 864 16282294

[B55] N'Guessan R Corbel V Akogbéto M Rowland M Reduced efficacy of insecticide-treated nets and indoor residual spraying for malaria control in pyrethroid resistance area, Benin Emerging Infect Dis 2007 13 199 206 10.3201/eid1302.060631 PMC2725864 17479880

[B56] Hargreaves K Koekemoer LL Brooke BD Hunt RH Mthembu J Coetzee M *Anopheles funestus *resistant to pyrethroid insecticides in South Africa Med Vet Entomol 2000 14 181 189 10.1046/j.1365-2915.2000.00234.x 10872862

